# Addressing Inequities in Access to Mental Healthcare: A Policy Analysis of Community Mental Health Systems Serving Minoritized Populations in North Carolina

**DOI:** 10.1007/s10488-024-01344-8

**Published:** 2024-01-29

**Authors:** Sasha Zabelski, Mara Hollander, Apryl Alexander

**Affiliations:** Department of Public Health Sciences, University of North Carolina, Charlotte, USA

**Keywords:** Health care Systems, Mental Health, Mental Health Services, Policy

## Abstract

Racial and ethnic minoritized uninsured populations in the United States face the greatest barriers to accessing mental healthcare. Historically, systems of care in the U.S. were set up using inadequate evidence at the federal, state, and local levels, driving inequities in access to quality care for minoritized populations. These inequities are most evident in community-based mental health services, which are partially or fully funded by federal programs and predominantly serve historically minoritized groups. In this descriptive policy analysis, we outline the history of federal legislative policies that have dictated community mental health systems and how these policies were implemented in North Carolina, which has a high percentage of uninsured communities of color. Several gaps between laws passed in the last 60 years and research on improving inequities in access to mental health services are discussed. Recommendations to expand/fix these policies include funding accurate data collection and implementation methods such as electronic health record (EHR) systems to ensure policies are informed by extensive data, implementation of evidence-informed and culturally sensitive interventions, and prioritizing preventative services that move past traditional models of mental healthcare.

## Background

Mental healthcare has faced numerous challenges over the last few decades. In 1963, President John F. Kennedy put forth a bill (Public Law 88–164) to dismantle state hospitals and expand community-based care for individuals with severe mental illness (SMI) through the establishment of community mental health centers (CMHCs; Erickson, 2021). Though initially these centers were created primarily to serve those with SMI, they expanded to serve individuals experiencing any mental health condition and offer inpatient, outpatient, partial hospitalization, crisis, and consulting services to the broader community (American Planning Associations, 1967). Since the enactment of Public Law 88–164, CHMCs have been funded through a combination of federal block grants, Medicaid, and CHIP reimbursements, individual state contributions, as well as Vocational Rehabilitation and Ticket to Work programs (Drake et al., 2016; Glied & Frank, 2006). Due to the various funding mechanisms, individuals who have Medicaid, are underinsured, or uninsured can receive services for free or on a sliding scale at CMHCs, though free/reduced service options are not consistent across states and even differ between CMHCs within the same state (Adams, 2023; Snowden & Thomas, 2000). Recently, funding has dwindled with 14% of CMHCs closing between 2014 and 2017 (Hung et al., 2020). Additionally, state block grants, which help sustain funding for many CMHCs, have not kept up with inflation leading to less funding distributed over time (Reich et al., 2017). Failing to sustainably fund these centers has resulted in an unmet need for mental health treatment, driving issues of access and diverting individuals to the criminal legal system, inpatient hospitalization, and homelessness (Rowan et al., 2013; Walker et al., 2015).

Issues with mental healthcare access are exacerbated at the intersection of race and insurance. Inaccessible CMHCs heavily impact communities of color, specifically Black, American Indian/Alaskan Native, and Latine populations. Studies have shown that Latine and Black people who are uninsured have decreased odds of accessing any type of mental healthcare and that expanding access to insurance reduces disparities in mental healthcare use for uninsured racially minoritized communities (Alegría et al., 2012, 2016). Urban low-income areas that are the catchment areas for most CMHCs serve a higher proportion of racial and ethnic minoritized communities (Chow et al., 2003; Cummings et al., 2017). With these issues in mind, it is vital to understand how policies can impact access to community-based care for minoritized communities by using a specific state as a case study for how legislation unfolds over time.

North Carolina was chosen as the focus of this analysis due to several reasons. Though North Carolina has had some progressive policy initiatives to enhance community-based services, the state still has one of the highest rates of persons unable to access mental healthcare in the U.S. (Mental Health America, 2022). Furthermore, North Carolina has a large percentage of underinsured/uninsured Black, Latine, and Native American communities who are more likely to have trouble accessing mental healthcare (KFF, 2021). Additionally, North Carolina has a significant number of people living in rural areas which are known to have fewer mental health resources and exacerbate issues related to access to care (Cline, 2023; North Carolina Rural Health Association, 2022). Therefore, we use North Carolina mental health policies as a case study to begin to understand how federal legislation leaves gaps for states to fill which if not fully done, can specifically harm communities that have the greatest need.

### Objective

Local, state, and federal policies govern how much money CMHCs receive and how they can use it in addition to prioritizing funding towards areas receiving the greatest public attention (e.g., suicide). In this descriptive policy analysis, we analyze both positive and negative aspects of mental health policies passed in Congress and in the North Carolina state legislature over the last 60 years and provide recommendations to close policy gaps that perpetuate disparities in mental healthcare access for underinsured/uninsured racial and ethnic minoritized communities.

## Methods

To compile policies for this review, we searched for federal laws using Congress.gov, filtering for “Laws” under “Legislative Actions.” We searched for North Carolina state laws using the North Carolina General Assembly website under the bill and laws section, specifically using the session law search. Our search included laws passed in 1963 and later, after the passing of the Community Mental Health Act. Keywords for both searches included “mental health” and “behavioral health.” Titles were screened to ensure that they were relevant to health. Due to this paper being a policy analysis as opposed to a systematic review, the exact number of article selections were not tracked, leading to potential missing legislation. We included legislation in this study if the law mentioned actions or changes to community mental health services. Laws were excluded if there was no specific mention of community mental health. For example, though the Americans with Disabilities Act (ADA) prohibited discrimination against individuals with SMI when it came to housing, employment, and other necessities, it did not specify the role of CMHCs in assisting individuals with SMI in this law; therefore, this bill was excluded from this analysis (U.S. Department of Justice, Civil Rights Division, n.d.). Legislation at the city and county level was not reviewed to keep this analysis broad; therefore, some laws passed at the granular levels may have been missed.

Additionally, current literature on the contributing factors to widening care gaps for marginalized populations were examined and evaluated in conjunction with legislative priorities identified in laws targeting community mental health services. Gaps were identified based on issues discussed in the mental health services literature around accessible healthcare that were not addressed in legislation. Recommendations were derived from reports and peer-reviewed literature discussing policy-based solutions to closing gaps in care.

### Federal Mental Health Policy Initiatives

The start of federal community mental health policy began with the Community Mental Health Act (Public Law 88–164) which helped develop the initial infrastructure of the CMHCs we know today (Erickson, 2021). Though this policy began the development of a community mental health system, many of the CMHCs that were going to be built as part of this legislation were never built partly due to budget cuts (highlighted below) resulting in a fragmented system (Erickson, 2021). The laws passed following the Community Mental Health Act, as outlined below, have since attempted to address these shortcomings but have failed to meet the needs of marginalized individuals experiencing mental health symptoms.

### Mental Health Systems Act

In 1980, Congress passed the Mental Health Systems Act which funneled grant money to nonprofit and for-profit CMHCs to research communities’ mental health needs, design programs to fit those needs, and involve communities in centers’ program development (Mental Health Systems Act, 1980). Funding focused on “mental health service which is” in an “area unserved or underserved by mental health programs,” (Mental Health Systems Act, p. 7, 1980) but with built-in restrictions around funding amounts, the system to service minoritized persons was already falling behind. The bill limited grants to $75,000 and only one grant was allowed to be awarded in each mental health service area. Both the Community Mental Health Act and the Mental Health Systems Act were almost immediately repealed due to federal budget cuts during the Reagan administration, resulting in few long-term impacts (Hunter, 1981). Specifically, much of the funding originally designated for the establishment of CMHC programs was converted into block grants putting funding responsibilities to the states and resulting in services shutting down (Estes & Wood, 1984).

### Affordable Care Act

The Affordable Care Act (ACA) was enacted in 2010 (Patient Protection and Affordable Care Act, 2010) to expand access to health insurance for millions of people at and below the poverty line. It originally required states to expand eligibility criteria for Medicaid, making more individuals eligible to be enrolled in Medicaid (U.S. Department of Health and Human Services, n.d.). In addition to expanding health insurance coverage for millions, the ACA had provisions focused on the integration of behavioral and physical health services, grants for training more mental health professionals, and added an option to Medicaid that allows the state to cover community-and home-based services for those with SMI (Patient Protection and Affordable Care Act, 2010). Following a 2012 Supreme Court decision (National Federation of Independent Business v. Sebelius, n.d.), states were granted the right to make the decision about whether to expand Medicaid. As of March 2023, there are 10 states who have decided *not* to expand benefits, most of them in the South (Kaiser Family Foundation, 2023a). As a result, the ACA failed to close gaps in states with communities who have lower access to care, states with a greater number of rural counties, states with large number of racially minoritized communities, and states with a greater percentage of uninsured individuals (Kaiser Family Foundation, 2023b). Medicaid is the biggest source of financial funding for CMHCs (Rosenbaum et al., 2019), but providers and staff are not able to tap into this funding source when patients are not eligible for Medicaid coverage. This is especially the case for states that choose not to enroll in the state covered Medicaid option that covers extensive home and community-based options.

### Mental Health Reform Act

In 2016, Congress passed the Mental Health Reform Act (Mental Health Reform Act of, 2016, 2016). This law focused on growing and maintaining the behavioral health workforce and establishing block grants for community mental health. With the passing of this law, the Substance Abuse and Mental Health Services Administration (SAMHSA) (established in 1992; SAMHSA, n.d.-a) became the primary survey administrator and reports the prevalence of accessing mental health and substance use services and treatment facility/client demographics for private and non-profit facilities. State-funding of community mental health services was also included in the bill with specific funding attention being brought to those with early symptoms of SMI and substance use disorders. Grants for crisis support services, support for integrated care, and prioritization of training mental health professionals to serve communities with the greatest need were all included. Overall, this bill clarified the prioritization of community-based mental health programming. However, the overall focus continues to be on grant applications that funnel money to services and communities that governmental agencies deem the most important rather than individual providers or nonprofit centers applying grant funding to what they feel is important to the communities they serve.

### CARES Act and American Rescue Plan Act

In the past, much of the legislation surrounding mental health treatment had focused on expanding insurance for mental health services and providing funding and grant opportunities for CMHCs. Recently, Congress passed two bills that specifically addressed issues of access, funding, and implementation of evidence-based care. The CARES Act of 2020 (CARES Act, 2020) expanded the use of telehealth for mental health services and ensured Medicaid and private insurance covered telehealth. The American Rescue Plan Act of 2021 (American Rescue Plan Act of 2021, 2021) provided funding for mental health training for health professionals/paraprofessionals and CMHCs, and continuing education opportunities for professionals working in behavioral health. It also addressed the lack of evidence-informed interventions provided in community mental health settings, including interventions addressing suicide and youth mental health, through specific funding dedicated to the development of evidence-informed programming and interventions.

#### Mental Health Policies Implemented in North Carolina

With each federal mental health policy passed, the government left room for individual states to fill in the gaps. States making their own decisions about the enactment of mental health policies resulted in varied and disjointed systems. North Carolina ranks lower than the national average on several social determinants of health (e.g., having higher levels of food insecurity, shortage of affordable housing; North Carolina Department of Health and Human Services [NCDHHS], n.d.-a); further exacerbating mental health struggles. For example, 33.5% of North Carolinians live below the federal poverty line, 12.9% are uninsured (compared to 10.4% of the national average), and only 3.3% of the population has Medicaid, while a higher proportion of residents also experience SMI symptoms and endorse poor health compared to the broader U.S. population (Kaiser Family Foundation, 2020). North Carolinians are also less likely to have a primary care doctor and choose to not use healthcare services due to cost. On a positive note, the number of uninsured persons in North Carolina is set to decrease with the recent passing of Medicaid expansion for the state potentially leading to a reduction in mental health disparities (Access to Healthcare Options, 2023).

Communities of color in North Carolina face greater disparities in rates of access to mental healthcare as compared to White populations. Only 31% of Black people and 4.7% of Latine individuals with SMI were served by the mental healthcare system compared with 63% of White individuals (SAMHSA, 2021). Yet, Black individuals make up 52% of psychiatric inpatient admissions, indicating that for this population, there are multiple barriers to community-based care such as discrimination, cost, and mistrust of the mental health system which prolongs seeking treatment and leads to escalation of mental health symptoms (Alang, 2019; Oluwoye et al., 2021; Satterfield, 2021). Latine/Hispanic, Black, and Native American people with SMI in North Carolina have a lower rate of accessing any type of mental healthcare compared to similar populations across the entire U.S. (SAMHSA, 2017). Individuals who lack insurance also have lower odds of accessing community-based care: North Carolinians without insurance make up a quarter of emergency department discharges for behavioral health diagnoses despite making up less than a fifth of the entire population (North Carolina Healthcare Association, 2022). Therefore, despite having similar rates of mental illness as compared to White populations (Panchal et al., 2022), racial and ethnic minoritized groups are at a disadvantage when it comes to accessing care. Due to the persistent disparities in access, it is vital to analyze whether mental health policy decisions made over the last 60 years in North Carolina perpetuated or reduced disparities in access for uninsured/underinsured racially and ethnically minoritized populations.

#### Executive Organization Act (1973)

In 1973, North Carolina passed the Executive Organization Act which created a Commission to govern prevention, treatment, and rehabilitation programs for mental health and substance use in North Carolina (Executive Organization Act of, 1973, 1973). Most of the Commission members are chosen by the Governor (24 members) with the last eight members being appointed by the General Assembly (NCDHHS-b, n.d.). Commission members are comprised of various stakeholders including service users, family members of service users, physicians, and attorneys (NCDHHS-a, 2023). The Commission works with the NCDHHS to pass rules that govern mental health service implementation (e.g., certifying and licensing facilities, NCDHSS-b, n.d.). With involvement from different members of the professional and service user community, the General Assembly was created to bring balance to decisions made by NCDHHS.

### SL 2001 − 437

In 2001 a significant mental health reform law was passed by the General Assembly in North Carolina requiring a reduction in the number of local mental health non-profit agencies to create broader catchment areas (39 areas reduced to 20 areas; Botts, 2002). Additionally, this bill required the NCDHHS to create a state plan targeted at helping underserved communities (Institute of Government, 2002). Each catchment area was required to provide data to NCDHHS about the number of people they served and the services those individuals received to understand broader gaps (Botts, 2006). This legislation aimed to create one system under which individuals receive care with a focus on interagency collaboration, evaluation, and continual improvement through county-wide evaluations. However, the legislation required the existing limited management entities (LMEs) to consolidate amongst themselves to create the broader catchment areas, in turn, reducing the number of services provided. This discrepancy was due to the merged catchment areas needing to use the same amount of state-funded money that was subdivided among more areas before the legislation passing to provide services to a greater number of individuals (Coates, 2016). This was further exacerbated in 2013 with the passing of SL 2011 − 264, requiring each catchment area to serve at least 500,000 people, leading to further consolidation of LMEs and fewer organizations serving bigger geographical areas (Botts, 2014). Advocates in North Carolina have argued that the move to using LMEs and consolidating service areas have decreased the number of services offered and made access to care more complicated (Knopf, 2023).

### American Rescue Plan Act Applied in North Carolina

The American Rescue Plan Act (ARPA) provided several behavioral health investments for states, including expansions of Certified Community Behavioral Health Clinics and additional block grants for community mental health services with North Carolina received more than $8 billion for these endeavors ($40 million of which were appropriated by SAMHSA via the Community Mental Health Services Block Grant) (NC Pandemic Recovery Office-a, n.d.). With these funds, the North Carolina government plans to expand crisis support services, bolster interventions for suicide prevention, purchase electronic health record (EHR) platforms for better integration of physical and behavioral health services, and integrate peer support specialists into more behavioral health settings (NC Pandemic Recovery Office-a, n.d.). Specifically, Governor Cooper’s push for an investment of $1 billion into the mental healthcare system and the bipartisan support for this plan led to an agreement in the use of this money (NC Pandemic Recovery Office-a, n.d.). The push for more equitable mental health service coverage during the pandemic led to the passing of Medicaid expansion in North Carolina and released incentive funds to the state that will be used to increase access to community mental health services (Robertson, 2023). However, with this being a more recent law, most funding initiatives have yet to be allocated and some of the initiatives that have been allocated (e.g. expansion of virtual behavioral health services) have yet to receive funding (NC Pandemic Recovery Office-b, n.d.). Therefore, the effects of this law on increasing access for marginalized communities is still unknown.

### Policy Gaps and Recommendations in Equitable Provision of Mental Health Treatment

Some strides have been made in expanding mental health policy to cover mental healthcare for historically minoritized communities. Sixty years of policy have included appropriations for CMHCs, expanding insurance coverage (including Medicaid), and broadening the use of telehealth for mental health service. States such as North Carolina have shined the spotlight on CMHCs as a primary provider of services to racial and ethnic minoritized populations by providing more targeted funding to maintain these services (e.g., SL 2001 − 437). This state-driven policymaking has allowed North Carolina to improve behavioral health systems.

However, substantial disparities continue to be present for communities of color, especially Black and Indigenous populations, which continue to face disproportionate rates of suicide and inaccessibility to care (Cénat, 2020; Stone et al., 2023; Thomeer et al., 2022). One driver of inequity is states choosing not to fund priority areas (e.g., only half of states have decided to use ARPA funding for the expansion of mobile crisis teams, which is the leading community-led alternative to law enforcement intervention in mental health calls; Guth, 2021) that significantly benefit minoritized uninsured populations (National Academies of Sciences, Engineering, and Medicine, 2017). Black and Latine people are more likely to receive inpatient services as opposed to community-based outpatient services (Alang & McAlpine, 2019; SAMHSA, 2021). Because Black individuals are more likely to have crises involving police due to systemic racism, the risk of being hospitalized is elevated (Chow et al., 2003). Black individuals are also more likely to ask for mental health services in jails and prisons and are less likely to have received treatment prior to incarceration compared to White individuals (Appel et al., 2020; Turner et al., 2023; Youman et al., 2010). In turn, mental distress from forced hospitalization through interactions with law enforcement reinforces the racism present in the mental health system that continues to drive Black communities away from seeking help (Legal Defense Fund & Bazelon Center for Mental Health Law, 2023; Shea et al., 2022).

By examining policy decisions made over the last 60 years and exploring the current literature on drivers of inequitable access, we concentrated on three specific pitfalls. Gaps include not enough legislative attention given to implementing an extensive mental health record system for better coordination of care, insufficient tracking of the administration of culturally sensitive services in community-based settings, and a greater fiscal focus on crisis care/inpatient beds than preventative services to provide care before symptoms escalate.

### Gap: Tracking Disparities in Service Use

Racial and ethnic disparities are further exacerbated when factoring in economic status, including lack of insurance (Maura & Weisman de Mamani, 2017). Many bills start the movement towards more equitable service provision for marginalized populations. For example, SL 2001-37 specifically focused on under-resourced areas (i.e., those with a disproportionate number of under/uninsured individuals struggling with SMI) and expanding and sustaining community behavioral health services for those areas through more funding. Understanding which communities are failing to receive services because of a lack of coordinated care after receiving an initial diagnosis/first encountering the mental health system (and why) can divert more resources to closing those care gaps and improving mental health outcomes. Unfortunately, as illustrated by several studies, community-based mental health services either do not extensively use systems for tracking data or insufficiently track mental health outcomes and service use (Alter et al., 2021; Bruns et al., 2016; Kariotis et al., 2022). One way to adequately understand differences in the type of resources needed for racially and ethnically minoritized populations is through consistent longitudinal data tracking such as electronic trails through the mental health and health systems.

### Recommendation: Statewide Data for Research and Implementation

Tracking a patient’s service use and related outcomes while using mental health services can help guide clinical and policymaking decisions to close gaps in inequitable access to care by understanding whether care continuity was achieved post-diagnosis (McGregor et al., 2015). Additionally, having standardized methods of using EHRs to document and subsequently improve gaps in care is vital, but rarely implemented in behavioral health settings (Kariotis et al., 2022). Only 23 states have plans to push forward some form of health information exchange (e.g., EHRs) for better system efficiency (Guth et al., 2023). Despite some EHR systems implemented at the state level over the last several years, there is still a significant gap in the collection of this data and how the data is being used to inform ways in which to improve mental health outcomes for underinsured/uninsured minoritized populations (CDC, 2023; Hoagwood et al., 2014). Furthermore, the lack of clear guidance on the use of these systems (e.g., use and satisfaction with services disaggregated by race, ethnicity, insurance status) has resulted in a patchwork of data reporting in each state and federal data tracking not providing enough data to make sustainable policy decisions (Hoagwood et al., 2014; NORC, n.d.; Predmore et al., 2023). A fragmented system of collecting data can result in missing diagnoses, gaps in follow-up care and can lead to errors in providing the right type of care (Madden et al., 2016).

Currently, EHR systems (similar to those used in physical health facilities) are not widely used in community-based services with many providers continuing to rely on paper records (Larrison et al., 2018; NCDHHS-b, 2023). Additionally, because of cost, smaller community-based agencies are less likely to use these systems to track patient data primarily (Larrison et al., 2018). Importantly, North Carolina has recognized a need for providing access to EHR systems to smaller/under resourced community centers by recommending Medicaid expansion funding be used on implementation of these systems, but the extent to whether this will be widely executed is still unknown (North Carolina Office of State Budget and Management, n.d.). Furthermore, having an integrated system through use of EHRs can streamline services and close gaps in access for minoritized groups.

### Gap: Smaller Emphasis on Funding Culturally-Informed Resources

Evidence-informed interventions, specifically created by and for racially and ethnically minoritized communities, are currently lacking in community-based settings. Culturally sensitive services not only focus on the needs of the population they are serving, but have staff trained and cognizant of how different aspects of culture (e.g., language, norms) are reflected in every step of the mental health treatment process (Guarnaccia & Rodriguez, 1996). There have been concerns about current evidence-based approaches, such as cognitive behavioral therapy (CBT), and their use with culturally diverse populations (Huey et al., 2023). According to the Centers for Disease Control and Prevention (CDC), creating accessible mental health resources would mean involving racially and ethnically minoritized individuals at all stages of an intervention including at the implementation stage (CDC, 2023). However, based on funding awards the last 2 years, provision of culturally informed services and culturally informed trainings for professionals have not been prioritized in North Carolina as only 7 out of 87 discretionary awards in 2023 and 2 out of 73 awards in 2022 have a grant aim related to providing culturally informed care (SAMHSA, n.d.-b). Therefore, prioritizing long-term funding for empirically informed interventions that are also supported by racially minoritized communities is essential.

### Recommendation: Funding Culturally-Informed Treatments for Minoritized Populations

Numerous studies have indicated the benefits of implementing culturally-informed care within mental health systems instead of “care as usual” (Garland et al., 2010; Weisz et al., 2012). Evidence-based interventions can also be cost efficient especially when considering the positive outcomes (e.g., decreased use of emergency departments; Levin & Chisholm, 2016; Moroz et al., 2020). Funding for these culturally-tailored interventions and specifically the implementation of interventions through training clinicians and updating supports to sustain these interventions is lacking. For example, Roundfield and Lang (2017) found that trauma-focused cognitive behavioral therapy (CBT) programs were not sustained for more than a few years in community-based mental health settings due to a lack of funding. In North Carolina, peer support services training models (specifically valued by Black, Indigenous, Latine people; Bakshi, 2021) recently received $4 million across 8 different agencies awarded through an ARPA block grant (NCDHHS, 2022). However, this money is to establish a pilot program and no further information has been found on how funds will be created to maintain this training model in the long-term.

Adequate implementation would also ensure that mental health clinicians and staff reflect the population they treat, and that every employee is trained on culturally responsive administration of interventions (CDC, 2023). Alternatively, provisions can be created to allow for promising practices for minoritized populations, but do not meet the criteria for culturally-informed treatments due to the lack of rigorous randomized clinical trials or funding. Most importantly, to ensure minoritized groups can access treatments built and implemented by individuals in their communities, funding of these programs need to be built into state/federal budgets.

### Gap: Not Enough Community Resources

Minoritized populations (e.g., racial and ethnic minoritized individuals from low-income backgrounds) experience high rates of SMI diagnoses but are less likely to access services. When they do receive help, they receive services in involuntary settings more so than their White counterparts (Mongelli et al., 2020). A lack of community resources in North Carolina was highlighted in a legislative report conducted by the NCDHHS. The authors note that there’s “an imbalance of community-based services relative to inpatient, residential, and institutional care in North Carolina, even though community-based services are often more cost effective” (NCDHSS, p.5, 2018). Additionally, the North Carolina Healthcare Association, who advocates on behalf of the state’s hospitals, recently included “expanded access to community-based behavioral health services with an emphasis on early intervention and treatment” to its list of legislative priorities (North Carolina Healthcare Association, 2022). Hay and colleagues (2022) discuss how the lack of access to primary care and community-based services in rural North Carolina affects individuals who live in poverty and note the importance of community partner engagement in assessing health needs and implementing culturally appropriate solutions. In addition, North Carolina has faced a behavioral health workforce shortage, contributing to individuals not receiving care in the community (Covino, 2019; Hay et al., 2022).

### Recommendation: Funding towards Outreach/Preventative Services

With evidence showing that uninsured Black and Latine people disproportionately access community-based care at lower rates than White people (Division of Diversity and Health Equity, 2017; Cook et al., 2016), policymakers should target populations that are rightfully distrusting of mental health systems and experience stigma around accessing services (Eylem et al., 2020; Fripp & Carlson, 2017). Reaching racial and ethnic minoritized communities prior to mental health symptoms becoming worse can reduce unnecessary hospitalizations.

Preventative services that incorporate persons with lived experience can help break down stigma, distrust, and White-driven narratives within community mental health (Bakshi, 2021). Current and past federal and state funding initiatives do not reflect the need to expand preventative and outreach services led by communities most impacted by systemic barriers to care. These types of services should involve: (1) funding Black, Latine, Native American, and other clinicians *and* non-clinicians of color to lead service development and provision (CDC, 2023); (2) envisioning other mental health treatment outside of the traditional therapy model (van Os et al., 2019); and (3) considerations for intersectionality in the development of mental health preventative/outreach programming (Oexle et al., 2018). For example, programs involving religious groups or churches that serve communities of color can fund non-traditional mental health roles (e.g., clergy) to provide outreach and support to improve mental health outcomes for these groups (Bellamy et al., 2021; Williams et al., 2014). Prioritizing these factors in funding initiatives can ensure that underinsured/uninsured racial and ethnic minoritized groups are receiving care created for them and administered by individuals who look like them. Additionally, by prioritizing policy initiatives that fund programming in community-based settings provided by professionals and lay persons of color, mental health systems can directly target well-known barriers to accessing care for racial and ethnic minoritized populations.

## Conclusion

Despite significant mental health policies enacted on the federal and North Carolina state level, gaps in accessible community mental health services persist for minoritized communities. Though there has been progress towards closing inequities in mental healthcare access, many laws that were passed to address the resource deficiency in CMHCs are new; therefore, there is uncertainty in the long-term impacts these laws may or may not have on marginalized communities (e.g., the effect of Medicaid expansion on access to CMHCs). Thus, by examining the legislation and studies analyzing disparities in access, we found gaps that include a lack of systematic data tracking and research, shortcomings in implementation of culturally sensitive evidence-informed treatments, and less funding focus on preventative/outreach services. To address these gaps, policy initiatives can focus on funding systems that can help with tracking mental health service coordination and outcomes, ensuring funding sustainability for culturally-tailored treatments, and supporting non-traditional professionals and programs to expand the reach of community-based resources. Future research should concetrate on how substantial funding policies can create sustainable community mental health services, the impact of differing amounts of funding on sustainability of CMHCs and how current policy initiatives may or may not help close disparities in access for minoritized communities in the long-term.
